# Efficacy prediction of neoadjuvant cadonilimab plus FLOT therapy for advanced gastric cancer: a study based on body composition changes

**DOI:** 10.3389/fonc.2025.1601819

**Published:** 2025-08-19

**Authors:** Penghui Liu, Na Li, Jizhen Wang, Lingyun Guo, Jiwu Guo, Guoqing Shi, Jie Mao

**Affiliations:** ^1^ Lanzhou University Second Clinical Medical College, Lanzhou, China; ^2^ Lanzhou University Second Hospital, The Medical Department, Lanzhou, China; ^3^ Lanzhou University Second Hospital, The General Surgery Department, Lanzhou, China

**Keywords:** neoadjuvant therapy, cadonilimab, FLOT, advanced gastric cancer, body composition, efficacy prediction

## Abstract

**Objective:**

This study aimed. to explore the predictive value of body composition changes in the efficacy of neoadjuvant cadonilimab combined with FLOT therapy for advanced gastric cancer and provide a reference for personalized treatment.

**Methods:**

A retrospective study was conducted on 33 patients with advanced gastric cancer who received neoadjuvant cadonilimab combined with FLOT therapy and subsequently underwent surgery. Body composition data were obtained using the InBody 720 body composition analysis device. Based on treatment response, patients were classified into the objective response group and the no-progression group. Quantitative data were presented as median and interquartile range. The Mann-Whitney U test was used for intergroup comparisons, analyzing the relationship between body composition changes before and after neoadjuvant therapy and treatment outcomes.

**Results:**

The changes in LBM, SLM, SMM, VFA, LEFT ARM SLM, RIGHT ARM SLM, LEFT LEG SLM, RIGHT LEG SLM, TRUNK SLM and IMPEDANCE before and after neoadjuvant therapy showed significant differences (P < 0.05) between two groups, indicating statistical significance. LBM, SLM and SMM showed a decreasing trend in both groups and the reduction was greater in the no-progression group than in the objective response group; VFA expressed a significant reduction in the objective response group, but it tended to increase in the no-progression group; IMPEDANCE showed a significant increase in the objective response group, but the change was insignificant in the no-progression group. SLM in the trunk and limbs showed a decreasing trend in both groups and the reduction was greater in the no-progression group than in the objective response group. The changes in HEIGHT, WEIGHT, BFM, PBF, LEFT ARM MBF, RIGHT ARM MBF, LEFT LEG MBF, RIGHT LEG MBF, TRUNK MBF, WHR and WAIST showed no significant differences (P ≥ 0.05) between two groups, indicating they were not statistically significant.

**Conclusions:**

The changes in LBM, SLM, SMM, VFA and IMPEDANCE can predict the efficacy of neoadjuvant cadonilimab plus FLOT therapy in advanced gastric cancer, especially LBM, SLM and SMM show the highest predictive value. Variations in SLM across different anatomical sites have distinct effects on treatment outcomes, the trunk has the most significant impact, followed by the lower limbs and the upper limbs have the least effect.

**Trial registration:**

www.chictr.org.cn, identifier ChiCTR2200066893.

## 1 Introduction

Gastric cancer is the fifth most common cancer and the third leading cause of cancer-related mortality worldwide, with high incidence and mortality rates (1). In East Asia, the burden of gastric cancer remains disproportionately high, accounting for nearly half of all global cases, underscoring the urgency of improving therapeutic strategies (2). Currently, substantial progress has been achieved in the chemotherapy, radiotherapy, immunotherapy and targeted therapy of gastric cancer. For patients with advanced gastric cancer, preoperative neoadjuvant therapy has become a crucial strategy for improving treatment efficacy and survival rates (3, 4). Among neoadjuvant regimens, the FLOT regimen, which consists of 5-fluorouracil (5-FU), leucovorin, oxaliplatin and docetaxel, has become a standard perioperative chemotherapy protocol in Western countries due to its superior pathological response rates and survival advantages over older regimens such as ECF (epirubicin, cisplatin and 5-FU) (5). In recent years, the combination of immunotherapy and chemotherapy has made significant progress in the treatment of advanced gastric cancer (6, 7). As a bispecific antibody targeting PD-1 and CTLA-4, cadonilimab has been clinically shown to markedly enhance pathological response rates and improve survival outcomes in patients with advanced gastric cancer when combined with FLOT chemotherapy (8). Although cadonilimab is a relatively novel agent, Long et al. have demonstrated in clinical trials that cadonilimab shows promising efficacy and manageable safety specifically in patients with advanced gastric cancer, supporting its potential use in the neoadjuvant setting (8). With the development of precision medicine, the prediction of patient treatment response has become an integral part of gastric cancer therapy. Accurately identifying patients who will benefit from specific therapies remains a major challenge due to the complex molecular and clinical heterogeneity of gastric cancer. Current predictive indicators mainly include clinicopathological factors such as tumor staging, histological type, tumor location and lymph node metastasis, as well as molecular biomarkers such as human epidermal growth factor receptor 2 (HER2), programmed death-ligand 1 (PD-L1) and microsatellite instability (MSI) (9–11). HER2 overexpression or gene amplification, which occurs in approximately 15–20% of gastric cancers, is associated with a better response to trastuzumab-based targeted therapy and serves as a critical biomarker for treatment stratification. PD-L1 expression, commonly assessed by combined positive score (CPS), helps identify patients likely to benefit from immune checkpoint inhibitors such as anti–PD-1/PD-L1 agents. MSI-high status, reflecting mismatch repair deficiency, has been linked to favorable prognosis and enhanced responsiveness to immunotherapy in gastric cancer (12). However, these markers still do not fully capture the heterogeneity of gastric cancer. Structural variants (SVs), including gene fusions, large insertions or deletions, and copy number alterations, play critical roles in driving tumorigenesis, promoting immune evasion, and mediating therapeutic resistance. In gastric cancer, SVs have been implicated in the dysregulation of oncogenes and tumor suppressor genes, and may contribute to intratumoral heterogeneity (13). Emerging evidence suggests that integrating SV profiling with established biomarkers could enhance the predictive accuracy of treatment response. However, their clinical utility is currently constrained by technical challenges in detection, limited standardization, and insufficient validation in large-scale prospective studies (14). While these markers provide some predictive value for treatment response, the heterogeneity of gastric cancer results in significant individual differences in treatment efficacy, single clinicopathological factor or molecular biomarker may not be sufficient to comprehensively and accurately predict the treatment response in gastric cancer patients. Consequently, exploring additional biomarkers has become a key strategy for enhancing the precision and personalization of gastric cancer treatment. In this context, multidimensional and non-invasive indicators such as body composition have gained increasing attention. As a critical biological indicator for evaluating cancer patients, body composition not only reflects tumor burden, nutritional status, and metabolic changes, but also plays a crucial role in monitoring treatment response. Some studies have shown that changes in body composition are closely associated with treatment response and prognosis in cancer patients (15–17). Nevertheless, body composition assessment is not yet routinely implemented in clinical decision-making for gastric cancer, partly due to the lack of standardized metrics and robust evidence in specific treatment settings such as neoadjuvant immunochemotherapy. Accordingly, this study aims to further explore the predictive value of body composition-related indicators in neoadjuvant therapy for advanced gastric cancer, hoping that it will provide new insights for evaluating the efficacy of neoadjuvant therapy.

## 2 Materials and methods

### 2.1 Study subjects

This study enrolled 38 patients with advanced gastric cancer, 5 patients were excluded due to missing data, leaving 33 for final analysis. Based on treatment response, patients were classified into two groups: the objective response group (15 cases) and the no-progression group (18 cases), The objective response group included patients with pathological complete response (pCR) and major pathological response (MPR). Inclusion criteria: (1) Pathologically confirmed advanced gastric cancer, meeting the clinical criteria for receiving neoadjuvant therapy. (2) All enrolled patients received treatment with Cadonilim plus the FLOT chemotherapy protocol. (3) Complete clinical data were available for all patients. (4) Body composition was measured before and after treatment using the InBody 720 body composition analysis device. Exclusion criteria: (1) Failure to complete the planned neoadjuvant therapy during the treatment period. (2) Missing body composition data, making effective analysis impossible. All participants voluntarily participated in the study and provided written informed consent. The study adhered to ethical guidelines and was approved by the relevant ethics committee.

### 2.2 Data collection

This study primarily collected patients’ demographic information, body composition data and treatment responses. All patients’ body composition were measured before and after treatment using the InBody 720 body composition analysis device. The body composition data included: height, weight, lean body mass (LBM), skeletal lean mass (SLM), skeletal muscle mass (SMM), body fat mass (BFM), percent body fat (PBF), visceral fat area (VFA), waist-to-hip ratio (WHR), waist circumference (WAIST) and impedance.

### 2.3 Statistical analysis

All statistical analysis were performed using SPSS software (version 26.0; IBM Corp., Armonk, NY, USA). Body composition changes for each parameter were calculated as the difference between post-treatment and pre-treatment values (Δ = Post-treatment value – Pre-treatment value). Data distribution was assessed visually and with the Shapiro–Wilk test. Due to non-normal distributions, quantitative variables were expressed as median (M) and interquartile range (IQR; P25–P75).

#### 2.3.1 Group comparisons

Baseline characteristics: Continuous variables (e.g., age, height, weight, BMI) were compared between the objective response group and no-progression group using the nonparametric Mann-Whitney U test. Categorical variables (e.g., gender, BMI categories) were compared using Pearson’s chi-square test.

Body composition changes: Differences in Δ values (e.g., ΔLBM, ΔSLM, ΔVFA) were analyzed using the Mann-Whitney U test between groups.

#### 2.3.2 Effect size and *post hoc* power analysis

For variables that did not reach statistical significance (e.g., ΔBFM, ΔPBF, ΔWHR), Cohen’s d was calculated to quantify the magnitude of differences. *Post hoc* power analysis was performed using G*Power software to assess the risk of Type II error, with statistical power (1-β) < 80% indicating insufficient power to detect true effects. Statistical significance was defined as a two-tailed *p-value < 0.05*.

## 3 Results

### 3.1 Comparison of baseline characteristics

This study found that pre-treatment BMI was significantly lower in the objective response group compared to the no-progression group (P < 0.05), indicating statistical significance. The results suggest that gastric cancer patients with lower BMI may have a better response to neoadjuvant therapy. Further categorical analysis of BMI revealed that although the difference between the two groups did not show statistical significance (p > 0.05), the clinical trend was informative. The proportion of normal-weight patients was significantly higher in the objective response group (86.67% vs. 61.11%), while the proportion of overweight and obese patients was higher in the no-progression group (38.89% vs. 6.67%). This result suggests that high BMI may be associated with decreased efficacy of neoadjuvant therapy in patients with advanced gastric cancer. AGE, HEIGHT, GENDER distribution and pre-treatment WEIGHT did not show significant differences between the two groups (p ≥ 0.05), indicating no statistical significance (**Table 1**).

**Table 1:** Baseline characteristics of 33 patients before treatment.

Indicators	Objective response group (n=15)	No-progression group (n=18)	*U/X^2^*	*p*
AGE [M (P25, P75)]	57.000 (48.0,68.0)	56.000 (46.5,61.5)	110.500	0.375
HEIGHT [M (P25, P75)]	170.000 (166.0,172.0)	170.000 (165.0,175.5)	127.000	0.769
WEIGHT (before treatment) [M (P25, P75)]	59.600 (57.5,65.6)	65.250 (59.1,80.6)	85.500	0.073
BMI (before treatment) [M (P25, P75)]	21.420 (20.2,22.8)	23.795 (21.6,25.3)	61.000	0.007**
GENDER [n (%)]			0.038	0.846
Female	2 (13.33)	2 (11.11)		
Male	13 (86.67)	16 (88.89)		
BMI (before treatment) [n (%)]			5.511	0.138
Underweight	1 (6.67)	0 (0.00)		
Normal weight	13 (86.67)	11 (61.11)		
Overweight	1 (6.67)	6 (33.33)		
Obese	0 (0.00)	1 (5.56)		

BMI, Body Mass Index. median (M) and interquartile range (IQR, P25-P75). n, number. **p<0.01.

### 3.2 Relationship between body composition changes and treatment efficacy

By analyzing the composition changes in 33 patients with advanced gastric cancer, the following results were obtained: The changes in LBM, SLM, SMM, VFA, IMPEDANCE, LEFT ARM SLM, RIGHT ARM SLM, LEFT LEG SLM, RIGHT LEG SLM and TRUNK SLM before and after neoadjuvant therapy showed significant differences (P < 0.05) between two groups, indicating statistical significance. The changes in WEIGHT, BFM, PBF, LEFT ARM MBF, RIGHT ARM MBF, LEFT LEG MBF, RIGHT LEG MBF, TRUNK MBF, WHR and WAIST showed no significant differences (P ≥ 0.05) between two groups, indicating no statistical significance (**Table 2**).

**Table 2:** Body composition changes indicators in 33 Patients.

Indicators	Objective response group M (P25, P75) (n=15)	No-progression group M (P25, P75) (n=18)	U	p
WEIGHT change	-6.400 (-9.4,-4.0)	-7.050 (-11.1,-0.9)	134.500	0.986
BFM change	-3.400 (-6.2,-0.5)	-2.600 (-5.8,3.5)	113.000	0.426
PBF change	-5.000 (-8.0,1.0)	-0.850 (-4.7,5.5)	96.500	0.164
LBM change	-3.100 (-4.2,-2.3)	-5.500 (-6.6,-4.8)	33.000	0.000**
SLM change	-2.900 (-4.0,-2.3)	-5.350 (-6.3,-5.0)	5.000	0.000**
SMM change	-1.700 (-2.4,-1.3)	-3.250 (-3.8,-3.0)	4.500	0.000**
VFA change	-51.000 (-56.0,-38.0)	12.000 (-18.3,41.5)	13.000	0.000**
IMPEDANCE change	105.000 (77.0,131.0)	24.000 (0.8,31.5)	5.000	0.000**
LEFT ARM SLM change	-0.220 (-0.3,-0.1)	-0.470 (-0.6,-0.3)	47.500	0.002**
RIGHT ARM SLM change	-0.230 (-0.3,0.0)	-0.545 (-0.6,-0.3)	34.500	0.000**
LEFT LEG SLM change	-0.420 (-0.6,0.1)	-0.800 (-0.9,-0.4)	76.000	0.033*
RIGHT LEG SLM change	-0.380 (-0.7,0.0)	-0.775 (-1.1,-0.5)	72.000	0.023*
TRUNK SLM change	-1.170 (-1.3,-1.0)	-2.600 (-3.1,-2.1)	4.000	0.000**
LEFT ARM BFM change	-0.220 (-0.4,-0.0)	-0.070 (-0.4,0.3)	103.000	0.247
RIGHT ARM BFM change	-0.220 (-0.3,0.0)	0.080 (-0.3,0.2)	104.500	0.270
LEFT LEG BFM change	-0.350 (-0.7,0.1)	-0.310 (-0.7,0.6)	122.500	0.651
RIGHT LEG BFM change	-0.420 (-0.7,-0.1)	-0.225 (-0.7,0.6)	105.500	0.286
TRUNK BFM change	-2.300 (-3.9,-0.9)	-1.250 (-3.0,1.1)	102.500	0.240
WHR change	-0.070 (-0.1,0.0)	-0.055 (-0.1,0.0)	116.000	0.490
WAIST change	-6.200 (-8.8,-2.8)	-4.200 (-9.8,4.7)	116.500	0.503

Data are presented as the median (M) and interquartile range (IQR, P25-P75). BFM, body fat mass; PBF, percent body fat; LBM, lean body mass; SLM, skeletal lean mass; SMM, skeletal muscle mass; VFA, visceral fat area; WHR, waist-to-hip ratio; WAIST, waist circumference; n, number. *p<0.05 **p<0.01.

### 3.3 Relationship between overall body composition changes and efficacy

**Figure 1:**
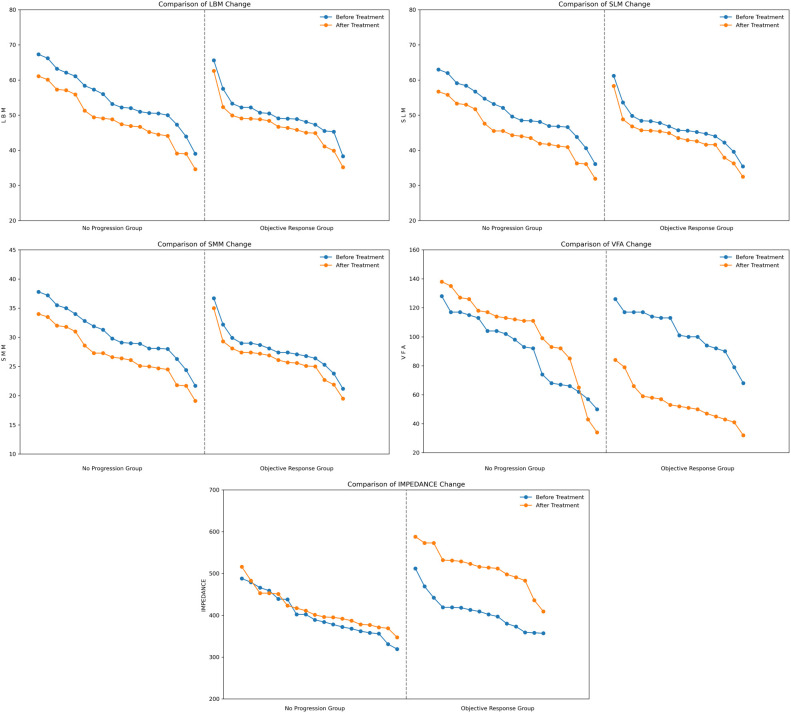
Comparison of overall body composition changes in 33 patients. LBM, lean body mass; SLM, skeletal lean mass; SMM, skeletal muscle mass; VFA, visceral fat area.

LBM change (no progression group = -5.500, objective response group = -3.100), SLM change (no progression group = -5.350, objective response group = -2.900), SMM change (no progression group = -3.250, objective response group = -1.700) before and after treatment. LBM, SLM and SMM showed a decreasing trend in both groups and the reduction was greater in the no-progression group than in the objective response group (**Figure 1**), suggesting that the more skeletal muscle loss, the less effective the treatment will be. VFA change (no progression group = 12.000, objective response group = -51.000) before and after treatment, VFA expressed a significant reduction in the objective response group, but it tended to increase in the no-progression group (**Figure 1**), suggesting that the more fat is reduced, the more effective the treatment will be. IMPEDANCE change (no progression group = 24.000, objective response group = 105.000) before and after treatment, impedance showed a significant increase in the objective response group, but the change was insignificant in the no-progression group (**Figure 1**), suggesting that the more impedance is increased, the more effective the treatment will be. LBM, SLM, SMM, VFA, and IMPEDANCE all showed significant differences between two groups (P < 0.01) (**Table 2**).

### 3.4 Relationship between localized body composition changes and efficacy

**Figure 2:**
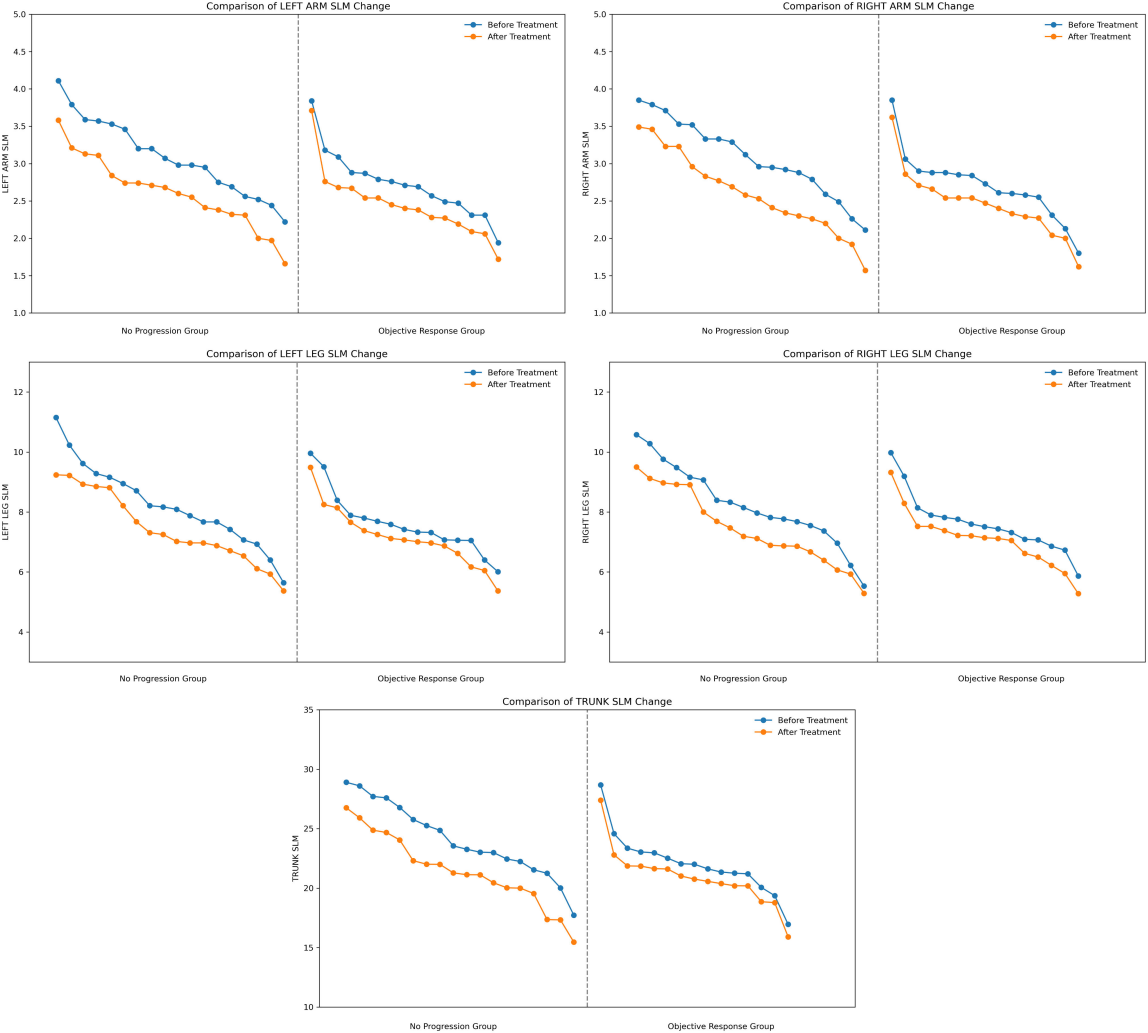
Comparison of localized body composition changes in 33 patients. SLM, skeletal lean mass.

In this study, we further analyzed the changes in SLM in different anatomical sites, including the trunk, right and left legs, right and left arms before and after treatment. The trunk SLM change was most distinct (no progression group = -2.600, objective response group = -1.170), followed by the lower limbs SLM change (no progression group: left leg = -0.800, right leg = -0.775; objective response group: left leg = -0.420, right leg = -0.380), and the upper limbs SLM change was smallest (no progression group: left arm = -0.470, right arm = -0.545; objective response group: left arm = -0.220, right arm = -0.230), SLM at all sites showed a tendency to decrease in both groups and the decrease was greater in the no-progression group than in the objective response group (**Figure 2**). Trunk SLM, Left Leg SLM, Right Leg SLM, Left Arm SLM, and Right Arm SLM all showed significant differences between two groups (P < 0.05) (**Table 2**).

### 3.5 Body composition changes reveal potential predictive biomarkers for immunotherapy efficacy

By integrating the visualized trends in **Figures 1** and **2** with the quantitative analysis results, we identified two innovative biological response patterns that can be used to guide the assessment of immunotherapy efficacy.

#### 3.5.1 The muscle-fat paradox: a dual-tissue marker reflecting treatment benefit

Patients in the objective response group showed distinct body composition adjustment trajectories, resulting in a bi-directional regulation characterized by “muscle preservation + fat loss”:

Protective retention of core muscles: Loss of key muscle indices (ΔLBM, ΔSLM, ΔSMM) was significantly attenuated in the objective response group (-1.7 to -3.1 kg), whereas muscle loss was more dramatic in the no-progression group (-3.25 to -5.5 kg).

Therapeutic visceral fat reduction: the objective response group showed a dramatic decrease in visceral fat area (ΔVFA: -51 cm²), whereas the no-progression group showed fat accumulation (ΔVFA: +12 cm²), suggesting a significant difference in metabolic pathway activation.

Impedance as a metabolic sentinel for tissue integrity: the significant increase in impedance in the objective response group (ΔIMP: +105 Ω vs. +24 Ω) may reflect the restoration of cellular structure, reduction of edema and improvement of metabolic status.

This dynamic composite biosignature of “muscle preservation + fat reduction” reveals a mechanism for predicting therapeutic benefit beyond traditional oncology metrics, with clear quantifiable advantages and clinical translational potential.

#### 3.5.2 Sensitivity and anatomical hierarchical pattern of trunk muscles

The distribution of sites of muscle reduction further reveals a spatially hierarchical pattern of response:

Trunk Dominant Loss: the reduction in trunk SLM in the no-progression group was 122% of that in the objective response group (-2.6 kg vs. -1.17 kg), suggesting that the mid-axis muscle groups are more sensitive to poor response to immunotherapy.

Distal gradient effect: Lower limb muscle loss was more significant (ΔSLM: -0.78 kg) than upper limb changes (ΔSLM: -0.51 kg), showing a decreasing response trend from trunk to limb.

In summary, preservation of trunk muscle mass and reduction of visceral fat can be used as key biomarkers for immunotherapy. Compared with traditional static pathologic indicators, these dynamic, noninvasive, repeatable body composition indicators can identify the trend of treatment response earlier, providing gastric cancer patients with a more accurate basis for efficacy prediction and treatment adjustment.

## 4 Discussion

Body composition indicators play a crucial role in assessing disease progression, treatment tolerance and prognosis in cancer patients. These indicators not only reflect the patient’s nutritional status and energy metabolism, but may also reveal changes in the tumor microenvironment and differences in response to treatment (18). It has been shown that significant muscle loss not only affects the body’s protein synthesis and immune function, but may also be associated with elevated levels of inflammation and imbalances in energy metabolism (19). Muscle loss may exacerbate cancer-related inflammation, elevated inflammatory factors such as interleukin-6 and tumor necrosis factor-α may lead to immunosuppression (20). This affects the effectiveness of treatment as well as reduces patient tolerance to neoadjuvant therapy, ultimately leading to tumor progression (21, 22). In this study, we found that LBM, SLM and SMM showed a decreasing trend in both groups of patients with advanced gastric cancer and the decrease was greater in the no-progression group, which suggests that muscle loss may be strongly associated with poorer treatment response, further validating the idea that muscle loss contributes to tumor progression. Ferreira et al. stated in a multicenter cohort study that localized low skeletal muscle density strongly associated with mortality in colorectal cancer patients (23). Our study found that in patients with advanced gastric cancer who received neoadjuvant cadonilimab combined with FLOT therapy, muscle changes at different anatomical sites all showed significant differences between the objective response group and the no-progression group, and SLM decreased most significantly in the trunk, followed by the lower limbs, with the least change in the upper limbs. The result suggests that there are differences in the sensitivity of different anatomical sites of muscle to neoadjuvant therapy in patients with advanced gastric cancer. Trunk muscles, especially core muscles, play an important role in maintaining metabolic homeostasis, supporting immune function and modulating anti-tumor inflammatory responses in the body, their significant reduction may be a key factor in the poorer response to treatment (24, 25). Reduction of limb muscles may reflect a decline in the patient’s overall physical status, the change can further affect patient’s tolerance of treatment and treatment outcome (26).

Adipose tissue affects the tumor microenvironment to some extent, Excess adipose tissue may promote tumor growth as well as affect immunotherapy outcome (27, 28). It has been shown that the reduction of adipose tissue can decrease insulin resistance and reduce the release of insulin-like growth factor, thereby inhibiting the activation of tumor cell proliferation signaling pathways (29). In addition, the reduction of adipose tissue may also affect the release of inflammatory factors such as interleukin-6 and tumor necrosis factor-α, thus improving the metabolic state of the body and the tumor microenvironment and increasing the sensitivity of treatment (30, 31). Our study found that VFA was significantly reduced in advanced gastric cancer patients in the objective response group, indicating that patients with greater VFA reduction were more likely to achieve pathological remission. The finding suggests that VFA reduction may play a positive role in promoting treatment response to neoadjuvant therapy in advanced gastric cancer. Our study also found that changes in BFM, PBF and localized fat did not reach statistical differences between two groups. The phenomenon may indicate that during neoadjuvant therapy for advanced gastric cancer, fat loss was concentrated in visceral fat, while changes in subcutaneous fat were more limited. However, the small sample size and limited representativeness may have affected the stability of the study results. Jayaprakasam et al. showed in their study that the response to neoadjuvant chemoradiotherapy in patients with locally advanced rectal cancer can be predicted using the changing characteristics of visceral fat (32), Our study results exhibit similarities to theirs, we further emphasized that changes in visceral fat may be a key factor in predicting the response to neoadjuvant therapy in advanced gastric cancer.

In recent years, the predictive value of nutritional and inflammatory status in cancer treatment has attracted considerable attention. Melekoglu et al. conducted a study in elderly patients with advanced gastric cancer undergoing perioperative FLOT therapy, using the modified Glasgow Prognostic Score (mGPS) to assess immunonutritional status. The results showed that elevated pre-treatment mGPS was significantly correlated with poorer overall survival, and that high BMI was an important risk factor for increased mGPS (33). This study suggests that fat accumulation may impair immunotherapy efficacy and survival outcomes in gastric cancer patients by exacerbating systemic inflammation and nutritional imbalance. Bayram et al. found that lower pre-treatment C-reactive protein/albumin ratio (CRP/Alb) and carcinoembryonic antigen/albumin ratio (CEA/Alb) were closely associated with higher pathological response rates in patients undergoing neoadjuvant therapy for gastric cancer. Specifically, a CRP/Alb ratio <2.74 and a CEA/Alb ratio <1.40 were associated with a 4.75-fold and 5.14-fold increase in the rate of pathological complete response (pCR), respectively. These findings suggest that lower systemic inflammatory burden and better nutritional status may enhance sensitivity to neoadjuvant therapy (34). In our study, we dynamically evaluated changes in fat and muscle composition during neoadjuvant therapy in patients with advanced gastric cancer from the perspective of body composition. We found that a significant reduction in visceral fat area (VFA) was associated with a higher pathological response rate. Our study also found that the pre-treatment BMI was significantly lower in the objective response group compared to the no-progression group. Moreover, a higher proportion of patients in the objective response group had a normal BMI, while the no-progression group had a higher proportion of overweight and obese patients. These findings are consistent with those reported by Melekoglu et al. These results suggest that adipose tissue is not merely a static nutritional indicator but may also dynamically modulate treatment response by influencing immune function, metabolic status, and the tumor microenvironment (35). From immunonutritional markers such as CRP, CEA, and albumin to the dynamic changes in body composition observed in our study, all highlight the critical role of host status on the efficacy of immunotherapy, providing valuable guidance for clinical practice. Therefore, integrating inflammatory scores with body composition indicators may help establish more precise and personalized models for predicting treatment efficacy, particularly for elderly patients or those at high nutritional risk. The validity and applicability of this integrated prediction pathway should be further validated in future multicenter studies.

Impedance reflects the conductive properties of the different components of human tissue such as water, fat, muscle and bone (36). In clinical practice, impedance is commonly used to assess a patient’s nutritional status and changes in body composition, especially fat and muscle distribution (37). Higher impedance usually means higher muscle density and relatively low fat content. Matthews et al. found perioperative impedance analysis may help predict risk of complications after elective cancer surgery (38), however, there are still fewer studies on the use of changes in impedance to predict cancer treatment outcomes. In this study, we found that the increase in impedance during neoadjuvant therapy in patients with advanced gastric cancer was significantly higher in the objective response group than in the no-progression group. The phenomenon suggests that changes in fat and muscle composition have an important impact on the outcome of neoadjuvant therapy, patients with higher muscle mass in advanced gastric cancer tend to respond more positively to neoadjuvant therapy.

This study is the first to systematically explore the relationship between body composition changes (such as muscle mass, fat distribution and impedance variation) and the efficacy of the cadonilimab combined with FLOT neoadjuvant therapy, providing new insights into the treatment of advanced gastric cancer. This innovative study design overcomes the limitations of traditional research that focus solely on tumor biological characteristics, highlighting the critical role of body composition in treatment response and providing a new perspective for optimizing neoadjuvant therapy strategies for advanced gastric cancer through body composition management. There are several limitations to this study: (1) The study included only 33 patients, the small sample size and insufficient representativeness may affect the stability and generalizability of the results. The small sample size increases the risk of type II error (failing to detect true differences), which may result in potentially important differences not reaching statistical significance and thereby obscure the relationship between body composition changes and treatment efficacy. For example, this study found that a reduction in visceral fat area (VFA) may positively influence the response to neoadjuvant therapy in gastric cancer. However, intergroup differences in the changes of BFM, PBF, regional fat, waist circumference, BMI and WHR were not statistically significant (P > 0.05). Considering the potential biological roles of these indicators in nutritional metabolism, immune regulation, and the tumor microenvironment, we further performed a *post hoc* power analysis using G*Power software. The results showed that the effect sizes of these variables were generally small to moderate, but the statistical power was substantially lower than the commonly recommended threshold of 80%, suggesting a potential risk of type II error. The current sample size may have been insufficient to detect the true impact of these variables on treatment response (**Table 3**). Therefore, the results of these variables that did not reach significance should be taken with caution, rather than being judged solely on the basis of the P-value as the sole criterion. Furthermore, intergroup comparisons were conducted using the Mann-Whitney U test, a non-parametric method that may exhibit limited statistical power with small sample sizes and is susceptible to the influence of extreme values, potentially increasing the risk of bias when interpreting group differences. Nevertheless, this study offers a novel perspective on predicting the efficacy of neoadjuvant immunochemotherapy by dynamically monitoring changes in body composition, which holds potential clinical relevance. In the future, multi-center and large-sample studies can be conducted to further validate the predictive value of body composition-related indicators on the effect of neoadjuvant therapy for advanced gastric cancer. (2) In the study, the InBody 720 body composition analyzer was used to measure body composition, it is highly reliable and easy to implement, however, there is still a gap compared to techniques such as CT imaging to measure muscle area or MRI to measure muscle density. Changes in body composition may be associated with various factors, including metabolic hormones and inflammatory cytokine levels, future studies could incorporate blood biomarkers to further explore the intricate mechanisms between metabolism, body composition and therapeutic response.

**Table 3:** Effect size and statistical efficacy analysis of selected non-significant variables.

Indicators	Objective response group (Mean ± SD)	No-progression group (Mean ± SD)	Cohen’s d	Power (1-β)	P
PBF change	-3.53 ± 5.82	-0.84 ± 7.11	0.215	0.091	> 0.05
BFM change	-3.07 ± 3.76	-1.98 ± 5.95	0.41	0.207	> 0.05
TRUNK BFM change	-2.09 ± 2.31	-1.29 ± 3.16	0.369	0.176	> 0.05
WAIST change	-5.55 ± 4.02	-3.32 ± 7.55	0.248	0.106	> 0.05
WHR change	-0.049 ± 0.052	-0.034 ± 0.068	0.289	0.126	> 0.05
BMI change	-2.061 ± 1.264	-2.185 ± 1.880	0.078	0.055	> 0.05

BFM, body fat mass; PBF, percent body fat; WHR, waist-to-hip ratio; WAIST, waist circumference; BMI, Body Mass Index; SD, Standard Deviation.

## 5 Conclusions

The results of this study indicate that changes in LBM, SLM, SMM, VFA and IMPEDANCE can predict the efficacy of neoadjuvant cadonilimab plus FLOT therapy in advanced gastric cancer, especially LBM, SLM and SMM show the highest predictive value. Variations in SLM across different anatomical sites have distinct effects on treatment outcomes, the trunk has the most significant impact, followed by the lower limbs and the upper limbs have the least effect. It remains necessary to validate these results in future studies with an expanded sample size.

## Data Availability

The original contributions presented in the study are included in the article/supplementary material. Further inquiries can be directed to the corresponding author.
